# Shape-Controlled Synthesis of Pt Nanopeanuts

**DOI:** 10.1038/srep31404

**Published:** 2016-08-16

**Authors:** Xuemei Zhang, Zengzilu Xia, Yingzhou Huang, Yunpeng Jia, Xiaonan Sun, Yu Li, Xueming Li, Rui Wu, Anping Liu, Xueqiang Qi, Shuxia Wang, Weijia Wen

**Affiliations:** 1 Soft Matter and Interdisciplinary Research Center, College of Physics, Chongqing University, Chongqing, 400044, P. R. China; 2 Department of Physics, The Hong Kong University of Science and Technology, Clear Water Bay, Kowloon, Hong Kong, China; 3 The State Key Laboratory of Power Transmission Equipment & System Security and New Technology, College of Chemistry and Chemical Engineering, Chongqing University, 400044, P. R. China

## Abstract

Exploring the novel shape of Pt nanoparticles is one of the most useful ways to improve the electrocatalytic performance of Pt in fuel cells. In this work, the Pt nanopeanuts consisting of two nanospheres grown together have been fabricated through a two-step polyol method. The high resolution scanning electron microscope (SEM) images and energy dispersive x-ray (EDX) spectrum collected at adjacent part point out the Pt nanopeanut is apparently different from the two physical attached nanospheres. To understand the growth mechanism of this nanopeanut, the final products in different synthesis situations are studied. The results indicate the interesting morphology of Pt nanopeanuts mainly benefit from the chemical reagent (FeCl_3_) while the size and homogeneity are greatly affected by the temperature. Furthermore, the electrocatalytic activity of the Pt nanopeanuts has also been demonstrated here. Our two-step synthesis of Pt nanopeanuts not only enlarges the group of Pt nanoparticles, but also provides a beneficial strategy for the synthesis of novel metal nanoparticles.

Noble metal nanoparticle has obtained a lot of interests in the last twenty years^1^ for their fantastic properties in optics^2^,^3^,^4^,^5^,^6^,^7^, electronics^8^,^9^,^10^,^11^,^12^,^13^,^14^, magnetics^15^, etc. Among them, Pt nanoparticle has been greatly focused on due to its excellent potentials applications in catalytic activity, which has great potential application in fuel cells and other related fields^16^,^17^,^18^,^19^. Since the catalysis activity is greatly influenced by the shape, size, material and configuration of Pt nanoparticles, lots of work have been made to acquire the high electrocatalytic performance, including manipulation the shape and size^20^,^21^, combining with other more abundant and less expensive material, changing the location on suitable surface^22^,^23^,^24^. Furthermore, a lot of Pt nanoparticles with various shapes and sizes have been reported, such as nanowires^25^, nanospheres^26^, nanocubes^27^. Although much effort has already been made in the field of shape controlling in the past^28^,^29^, the exploring of novel Pt nanoparticle synthesized by new method is still the core task of great scientific significance and commercial value now.

Herein, the work reports a novel structure of Pt nanopeanuts synthesized through a two-step polyol method. The high resolution scanning electron microscope (SEM) images and energy dispersive x-ray (EDX) data demonstrate this Pt nanopeanut is apparently distinguished from two physically attached individual Pt nanospheres. The results point out the reaction conditions including reaction temperature and chemical agent in the synthesis process play great impacts on the size and morphology of final products. And the electrocatalytic results of these Pt nanopeanuts demonstrate its potential application in energy field of fuel cells.

## Results and Discussion

### Synthesis of Pt nanopeanuts

In a typical two-step synthesis, 1 mL of 0.02 M FeCl_3_ aqueous solutions was firstly added into 20 mL ethylene glycol in a 100 ml round bottom flask with magnetic stirring for 5 minutes. Then, 1 mL of 0.05 M H_2_PtCl_6_ aqueous solutions was added. After stirring uniformity about 30 minutes at room temperature, the solution was heated for four hours in an oil bath at 100 °C (the reaction temperature at first step). And the temperature was raised to 180 °C and heated for another five and a half hours (the reaction temperature at second step). During the heating, the color of the solution changed from yellow to colorless and then, gradually, back to yellow and finally light grey. The final product could be obtained by centrifugal washing in ethanol.

### Physical characterization of the Pt nanopeanuts

**Figure 1:**
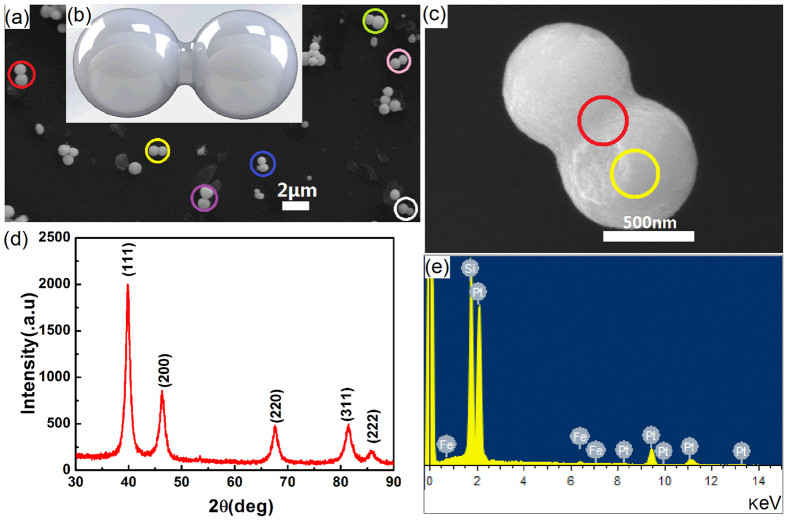
Physical characterization of the Pt nanopeanuts. (**a**) SEM image of Pt nanopeanuts indicated by color circles. (**b**) Sketch of Pt nanopeanut. (**c**) SEM image of a single nanopeanut. XRD (**d**) and EDX (**e**) spectrums of Pt nanopeanuts.

As indicated by the colored circles in SEM image in Fig. 1a, there are Pt nanopeanuts generated by the two-step synthesis method described above. The Pt nanopeanut consists of two nanospheres grown together but not physically atteched nanodimer, whose configuration is illustrated by the sketch in Fig. 1b. This novel configuration of Pt nanopeanut is further confirmed by the high resolution SEM image of single Pt nanopeanut shown in Fig. 1c. This SEM image points out this single Pt nanopeanut consists of two nanospheres with 600 nm diameters and the adjacent part shows clearly that the two nanopheres are grown together but not physical attached. Fig. 1e shows the EDX spectra, which collected at the adjacent part (red circle) and the center of nanosphere (yellow circle) respectively in Fig. 1c. The result exhibits obvious structure feature of Pt element, which also provides a strong support to the Pt nanopeanut but not Pt nanoparticle dimer. Figure 1d presents the typical Powder X-ray Diffraction (XRD) patterns of the prepared nanopeanuts. In the XRD spectrum, there are five diffraction peaks located at 39.76°, 46.23°, 67.45°, 81.26° and 85.69°, which could be indexed to (111), (200), (220), (311) and (222) planes of face-centered cubic crystalline Pt, respectively [JCPDS standard 89–7382(Pt)].

### The shape-controlled synthesis of Pt nanopeanuts

**Figure 2:**
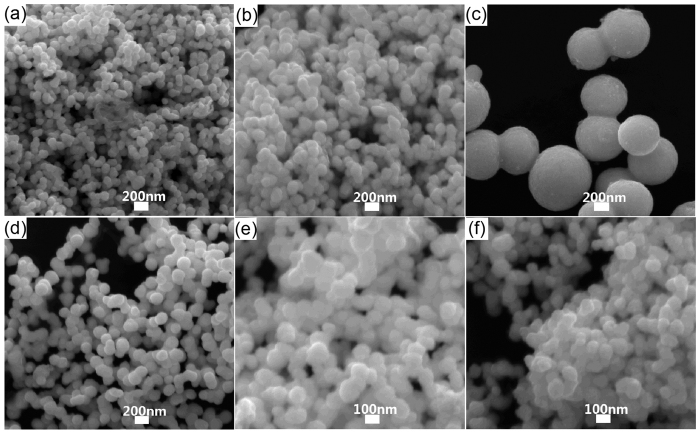
The effect of the temperature factors on forming Pt nanopeanuts. SEM images of Pt nanoparticles at different first reaction temperature (**a**) 60 °C, (**b**) 80 °C, (**c**) 100 °C, (**d**) 120 °C, (**e**) 140 °C, (**f**) 160 °C.

**Figure 3:**
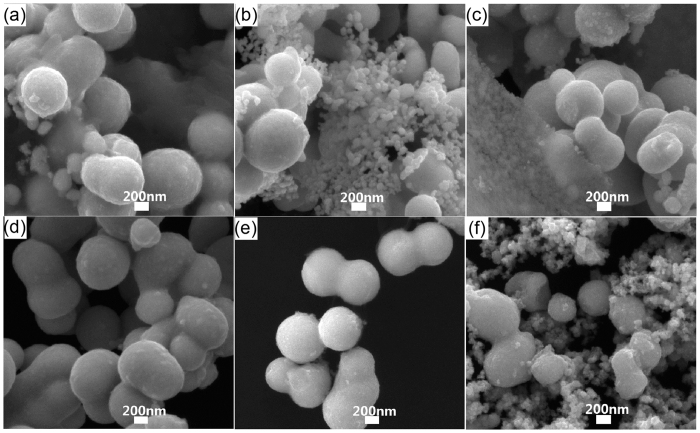
The effect of the temperature factors on forming Pt nanopeanuts. SEM images of Pt nanoparticles at different second reaction temperature (**a**) 100 °C, (**b**) 120 °C, (**c**) 140 °C, (**d**) 160 °C, (**e**) 180 °C, (**f**) 200 °C.

To investigate the growth mechanism, the influence of temperature and chemical agent on the synthesis of Pt nanopeanuts are studied. As introduced above, there are two steps in a typical Pt nanopeanut synthesis including primarily maintaining at 100 °C for four hours (the first step) and then staying at 180 °C for five and a half hours (the second step). To achieve controlling over the shape and size of final product, the influence of reaction temperature in two steps is studied respectively. First, the SEM images of generated Pt nanoparticles at different reaction temperature in first step are shown in Fig. 2, where the first reaction temperature varies from 60 °C to 160 °C while the second reaction temperature remains 180 °C. Obviously, the Pt nanoparticles in these SEM images exhibit greatly difference in size that the Pt nanopeanuts at 100 °C in Fig. 2c have much greater diameters compared to the Pt nanoparticles at other temperature about 100 nm in Fig. 2a,b or d–f. And then the SEM images of final product at different reaction temperature in second step are shown in Fig. 3, where the second reaction temperature varies from 100 °C to 200 °C while the first reaction temperature remains 100 °C. Obviously, the Pt nanoparticles with about 600 nm diameters are generated in all condition in Fig. 3. And the Pt nanopeanuts with high homogeneity are obtained at 180 °C. However, the by-product of much small Pt nanoparticles are also induced at temperature lower than 160 °C (Fig. 3a–c) or higher than 180 °C (Fig. 3f).

Therefore, the reaction temperature in this two-step synthesis plays an important role in the size and homogeneity of Pt nanopeanuts. The much greater diameter in first step at 100 °C (Fig. 2c) might indicate the majority of reduced Pt atoms choose to adsorb on the surface of Pt precursors, which makes the existing precursors keep growing bigger. However, the existing precursors stop growing under a maximal size at other first reaction temperatures, which makes reduced Pt atoms tend to generate new Pt precursors. The appearance of small Pt nanoparticles in Fig. 3 are probably because the reduced Pt atoms prefer to generate new precursors rather than adsorb on the existing precursors in second step (Fig. 3e).

**Figure 4:**
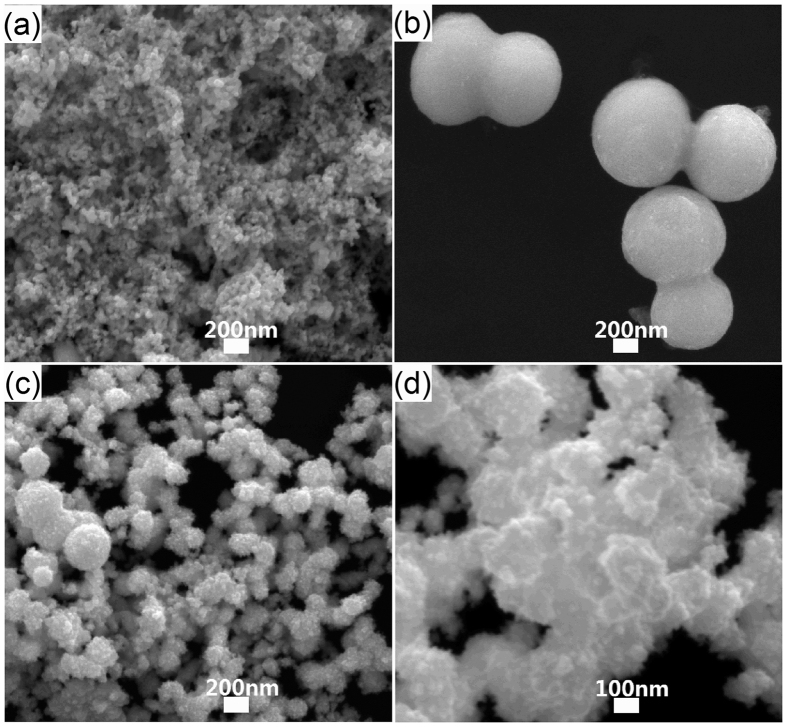
The effect of the FeCl_3_ concentration factors on forming Pt nanopeanuts. SEM images of Pt nanoparticles with (**a**) no, (**b**) 0.02 M, (**c**) 0.04 M, (**d**) 0.06 M FeCl_3_.

Furthermore, the influence of the FeCl_3_ concentration on the Pt nanoparticle shape is also investigated in Fig. 4, where the final products are quite different. Without FeCl_3_, the small Pt nanoparticle with a mean diameter less than 20 nm is obtained (Fig. 4a). However, with the increase of FeCl_3_ concentration, the spikes were clearly formed on the surface of the Pt nanparticles (Fig. 4c,d). It is very clear in Fig. 4b that the Pt nanopeanuts with smooth surface are fabricated at 0.02 M FeCl_3_. According to Xia’s reports^30^,^31^,^32^, a trace amount of iron species (FeII or FeIII) in a polyol process could significantly alter the growth kinetics of Pt nanostructures and induce the formation of Pt nanowires, which could be the main reason for the synthesis of Pt nanopeanut here. The generation of nanowire on Pt nanoparticle might enhance the probability of Pt nanoparticle aggregating. With a small amount of iron species, there is little nanowire at Pt precursor surface that neighbor two precursors partly merge together and form nanopeanut with the following reduced Pt atom. Nevertheless, too much nanowire on precursor surface makes much more precursors grown together to generate nanoparticle aggregating but not nanopeanuts. Therefore, the data in Fig. 4 demonstrate the great significance of FeCl_3_ in the Pt nanopeanut synthesis.

**Figure 5:**
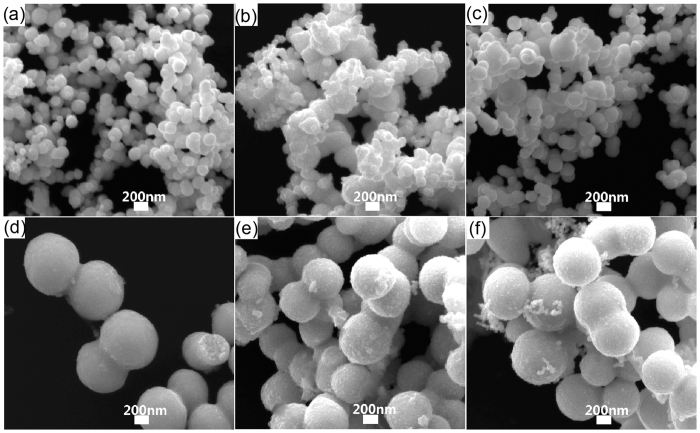
The effect of the H_2_PtCl_6_ concentration factors on forming Pt nanopeanuts. SEM images of Pt nanoparticles with (**a**) 0.02 M, (**b**) 0.03 M, (**c**) 0.04 M, (**d**) 0.05 M, (**d**) 0.06 M, (**d**) 0.07 M H_2_PtCl_6_.

Besides FeCl_3_, our results also point out the H_2_PtCl_6_ concentration could play great impact on the growth of the Pt nanoparticles. As shown in Fig. 5, the main final product at a low H_2_PtCl_6_ concentration (<0.05 M) is Pt nanoparticle aggregating consist of nanoparticle with diameter about 100 nm (Fig. 5a–c). And the byproduct of small nanoparticle with diameter about 20 nm (Fig. 5e,f) is obtained at a high H_2_PtCl_6_ concentration (>0.05 M).

### The electrocatalytic activities of the Pt nanopeanuts

**Figure 6:**
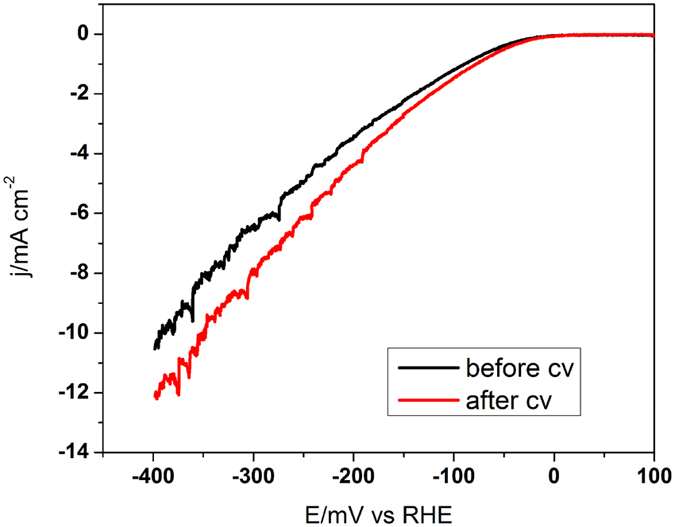
The electrocatalytic activities of the Pt nanopeanuts. The comparative LSV curves of Pt nanopeanuts in N_2_-saturated 0.1 M KOH electrolyte.

To acquire the practical application of the as-prepared novel Pt nanopeanuts, electrochemical measurement has been performed to study their catalytic activity towards the hydrogen evolution reaction (HER). As shown in Fig. 6, liner sweep voltammetry (LSV) ranges from −0.4 to 0.1 V vs. Reversible hydrogen electrode is carried out at a scan rate of 50 mV s^−1^ in 0.1 M KOH. The nanopeanuts exhibit a certain electrocatalytic activity for HER. The onset potential of the Pt nanopeanuts is close to 0 mV, the current density achieves to 10 mA cm^−2^ at the overpotential of 400 mV, and it exhibits an increased current density with a value of 12 mA cm^−2^ after 1000 CV cycles at the same potential. The HER test demonstrates that the as-prepared Pt nanopeanuts have the potential applications for the electrochemistry.

## Conclusions

The Pt nanopeanut consisting of two nanospheres grown together has been fabricated through a two-step polyol method, which is confirmed by the high resolution SEM image and EDX spectrum obtained at the adjacent part. Further studies point out both the reaction temperature and the concentration of FeCl_3_ play key roles in the generation of the Pt nanopeanuts. The electrocatalysis test figures out this novel Pt nanopeanuts has potential application in the fields of fuel cells. Our two-step polyol method for Pt nanopeanuts not only enlarges the group of Pt nanoparticles but also present a useful route for the synthesis of novel metal nanoparticle including Au, Ag, Pt, Pd, etc.

## Methods

### Electrochemical measurement

he nanopeanuts were evaluated electrochemically in three-electrode one-compartment cell, in which 0.1 M KOH was used as the electrolyte. The electrocatalytic activity of nanopeanuts for HER was determined by LSV at a sweep rate of 10 mVs^−1^ with a rotational disk electrode (RDE). The measurements were performed at 25 °C after purging N_2_ for 30 min. RDE measurements were conducted with a varying rotating speed of 1600 rpm. Here a glassy carbon disk of 5 mm diameter coated with a film of nanopeanuts was used as working electrode. To prepare the working electrode, the prepared nanopeanuts and a drop of 0.1 wt% Nafion solution were ultrasonically dispersed in 200 mL of a water-ethanol (1:1 v/v) mixed solvent to form a homogeneous ink. Then 12 μL of the catalyst ink was loaded onto a RDE. After evaporation of ethanol in air, the catalyst layer was covered with a thin film of Nafion by adding a drop of 0.01 wt % Nafion solution. Finally, the RDE was dried at air temperature.

## Additional Information

**How to cite this article**: Zhang, X. *et al*. Shape-Controlled Synthesis of Pt Nanopeanuts. *Sci. Rep.* **6**, 31404; doi: 10.1038/srep31404 (2016).
